# Bridging the distributional gap of *Tylorida striata* (Thorell, 1877) and new synonymy (Araneae: Tetragnathidae)

**DOI:** 10.3897/BDJ.3.e4878

**Published:** 2015-03-26

**Authors:** Siddharth Kulkarni, Swara Yadav

**Affiliations:** ‡ Biome Conservation Foundation, Pune, 18, Silver Moon Apts.,1/2A/2, Bavdhan Kh.,Pune 411021, India; § Zoology Department, Yashavantrao Chavan Institute of Science, Satara, Satara, Maharashtra, India

**Keywords:** *
Tylorida
*, syntypes, synonymy, new record, wide distribution

## Abstract

**Background:**

Although *Tylorida striata* has not been reported from India, observations on India Biodiversity Portal (IBP 2015), an open access repository for biodiversity information of Indian subcontinent, showed images resembling this species. The respective locality in Gujarat, India was explored and specimens were studied to confirm record of *T. striata* in India. Literature study showed some taxonomic lacunae which needed to be resolved.

**New information:**

The tetragnathid spider *Tylorida striata* (Thorell, 1877) is redescribed on examination of its long unknown syntypes from Kendari, Southeast Sulawesi deposited in the Natural History Museum of Giacomo Doria, Genoa, Italy and additional material from Kendari and other Indonesian regions. Description of *Tylorida stellimicans* (Simon, 1885) mentions its identity with *T. striata*, which however characterizes sub-adult of *T. striata* and is accordingly synonymized. *Tylorida striata* is newly recorded from India which links the distributional gap between China to Australia and Comoros Islands. Distributional and taxonomic records of *Tylorida* species with *T. striata*- like globose abdomen infer probable synonymy, which is subject to genus revision.

## Introduction

The World Spider Catalog (2015) currently consists of ten accepted *Tylorida* species distributed in parts of Asia, Australasia and Africa. Simon (1894) established genus *Tylorida* on the basis of *Meta striata* Thorell, 1877. Currently valid as *Tylorida striata* (Thorell, 1877), this species has a long and interesting taxonomic and faunistic history. In a series of publications on spiders from Malaysia and Papua New Guinea, Thorell (1877) described *Meta striata* based on female specimens collected from Kandari (now Kendari), South east Sulawesi, Indonesia mentioning this species is also found on Amboina (now Ambon Islands), Indonesia. Hasselt (1882) described its male from Sumatra. Strand (1922) reported a male from Bandar Bahive, Sumatra. He noted that it is not easy to identify this species on the basis of previous literature and warned that, difficulty in understanding its femoral setae could lead to its placement in wrong genus. Gerhardt (1923) nicely illustrated the male palp showing the coiled spermatic ducts.

Bösenberg and Strand (1906) described *Tylorida magniventer* from an unknown location in Japan which was later synonymised with *T. striata* by Tanikawa (2005). *Tylorida striata* has been reported a number of times from Japan (Yaginuma (1960), Yaginuma (1971), Yaginuma (1986)). Kulczyński (1911) reported the species from Andai, North New Guinea based on a female specimen; Chrysanthus (1963), Chrysanthus (1975) reported the species based on a female from South New Guinea; Lee (1966) reported a male from Taiwan; Davies (1988) illustrated female epigynum and male palp using Australian specimens.

Dimitrov et al. (2008) examined male holotype of *Sternospina concretipalpis* Schmidt & Krause, 1993 from the Comoros Islands and synonymised with *T. striata*, thus extending the species distribution far to the west. They noted conspicuous distributional gap and speculated that it may be due to lack of study, particularly from the Indian subcontinent. A detailed morphological description and phylogenetic position of *T. striata* in reference to its genus was provided in the atlas of Tetragnathidae (Álvarez-Padilla and Hormiga 2011). However, the whereabouts of Thorell's type(s) of *Meta striata* were unknown (Álvarez-Padilla and Hormiga 2011, Dimitrov et al. 2008).

## Materials and methods

Syntypes of *Meta striata* were loaned from Natural History Museum of Giacomo Doria, Genoa, Italy (MSNG) at Naturalis Biodiversity Center, Leiden, The Netherlands for examination by Siddharth Kulkarni and imaged using Nikon DS-Ri1 mounted on Leica M165C™ stereozoom microscope, Indonesian material using Zeiss™ Stemi SV11 stereomicroscope and Indian material using Brunel IMXZ™stereozoom microscope and imaged using Canon 1200D™ mounted camera. Indonesian specimens are deposited at Naturalis Biodiversity Center (RMNH), Leiden, The Netherlands and Indian specimens are deposited at Bombay Natural History Society (BNHS), Mumbai. Map was prepared using QGIS (2015). All morphological measurements are in millimetres (mm).

## Taxon treatments

### Tylorida striata

(Thorell, 1877)

853163

853158

Meta striata Thorell, 1877: 427.
Meta striata Hasselt, 1882: 25.
Argyroepeira bigibba Thorell, 1887: 140.
Argyroepeira *s.* Thorell, 1887: 142.
T. striata Simon, 1894a: 737, f. 809.
Argyroepeira *s.* Workman & Workman, 1894: 19, pl. 19.
Tylorida magniventer Bösenberg & Strand, 1906: 187, pl. 15, f. 397.
T. striata Bösenberg & Strand, 1906: 187, pl. 15, f. 420.
Sternospina concretipalpis Schmidt and Krause, 1993: 7, f. 1.
Tylorida stellimicans Simon, 1885: 449. **New synonymy.**

#### Materials

- **Type status:** Syntype. **Occurrence:** recordedBy: O. Beccari; individualCount: 2; sex: Female; lifeStage: adult; occurrenceStatus: present; **Taxon:** scientificNameID: urn:lsid:nmbe.ch:spidersp:014401; scientificName: Tylorida striata; kingdom: Animalia; phylum: Arthropoda; class: Arachnida; order: Araneae; family: Tetragnathidae; genus: Tylorida; taxonRank: species; scientificNameAuthorship: (Thorell, 1877); nomenclaturalCode: ICZN; taxonomicStatus: accepted; **Location:** locationID: Ambon Islands, Indonesia; continent: Asia; islandGroup: Maluku archipelago; island: Ambon; country: Indonesia; countryCode: IDN; **Identification:** identifiedBy: Thorell; identificationReferences: Studi sui Ragni Malesi e Papuani. I. Thorell, T. (1877); **Event:** year: 1874; **Record Level:** type: specimen; institutionID: Museo Civico di Storia Naturale “Giacomo Doria”, Italy; institutionCode: MSNG; basisOfRecord: Preserved specimen
- **Type status:** Other material. **Occurrence:** recordedBy: C.L.and P.R. Deeleman; individualCount: 1; sex: 1 female; **Taxon:** taxonID: urn:lsid:nmbe.ch:spidersp:014401; scientificName: Tylorida striata; kingdom: Animalia; phylum: Arthropoda; class: Arachnida; order: Araneae; family: Tetragnathidae; genus: Tylorida; taxonRank: species; scientificNameAuthorship: (Thorell, 1877); **Location:** locationID: TYPE LOCALITY; continent: Asia; country: Indonesia; countryCode: IDN; stateProvince: SOUTH EAST SULAWESI; locality: Taneah, 40 km south of Kendari, sea-faced; **Event:** eventDate: 08/11/1980; **Record Level:** type: specimen; institutionID: Naturalis Biodiversity Center, Leiden; institutionCode: RMNH; collectionCode: Arachnida; basisOfRecord: PreservedSpecimen
- **Type status:** Other material. **Occurrence:** catalogNumber: BNHS Sp. 139–144; occurrenceRemarks: live in its orb web; recordedBy: Prathamesh Patel; individualCount: 6; sex: 6 females; lifeStage: adult; occurrenceStatus: present; **Taxon:** scientificNameID: urn:lsid:nmbe.ch:spidersp:014401; scientificName: Tylorida striata; kingdom: Animalia; phylum: Arthropoda; class: Arachnida; order: Araneae; family: Tetragnathidae; genus: Tylorida; taxonRank: species; scientificNameAuthorship: (Thorell, 1877); nomenclaturalCode: ICZN; taxonomicStatus: accepted; **Location:** locationID: 853155; continent: Asia; country: India; countryCode: IN; stateProvince: Gujarat; locality: Pariyer; verbatimCoordinateSystem: decimal degrees; decimalLatitude: 22.56; decimalLongitude: 72.954; **Identification:** identifiedBy: Siddharth Kulkarni; identificationReferences: Studi sui Ragni Malesi e Papuani. I. Thorell, T. (1877); Morphological and phylogenetic atlas of the orb-weaving spider family Tetragnathidae. Álvarez-Padilla, F.; **Event:** samplingProtocol: visual searching; eventDate: 2014-07-09; year: 2014; month: 7; day: 9; habitat: Low grass; eventRemarks: post monsoon; **Record Level:** type: specimen; institutionID: Bombay Natural History Society, Mumbai; collectionID: Spider; institutionCode: BNHS; collectionCode: Sp.; basisOfRecord: PreservedSpecimen
- **Type status:** Other material. **Occurrence:** catalogNumber: BNHS Sp. 154-157; occurrenceRemarks: live in its orb web; recordedBy: Atul Vartak; individualCount: 4; sex: 4 females; lifeStage: adult; occurrenceStatus: present; **Taxon:** scientificNameID: urn:lsid:nmbe.ch:spidersp:014401; scientificName: Tylorida striata; kingdom: Animalia; phylum: Arthropoda; class: Arachnida; order: Araneae; family: Tetragnathidae; genus: Tylorida; taxonRank: species; scientificNameAuthorship: (Thorell, 1877); nomenclaturalCode: ICZN; taxonomicStatus: accepted; **Location:** continent: Asia; country: India; stateProvince: Maharashtra; locality: Boisar; verbatimCoordinateSystem: decimal degrees; decimalLatitude: 19.8; decimalLongitude: 72.75; **Identification:** identifiedBy: Siddharth Kulkarni; identificationReferences: Studi sui Ragni Malesi e Papuani. I. Thorell, T. (1877); Morphological and phylogenetic atlas of the orb-weaving spider family Tetragnathidae. Álvarez-Padilla, F.; **Event:** samplingProtocol: visual searching; eventDate: 2014-10-10; year: 2014; month: 10; day: 10; habitat: Low grass; eventRemarks: post monsoon; **Record Level:** type: specimen; institutionID: Bombay Natural History Society, Mumbai; collectionID: Spider; institutionCode: BNHS; collectionCode: Sp.; basisOfRecord: PreservedSpecimen
- **Type status:** Other material. **Occurrence:** recordedBy: C.L and P.R. Deeleman; individualCount: 5; sex: 4 females, 1 juvenile; **Taxon:** taxonID: urn:lsid:nmbe.ch:spidersp:014401; scientificName: Tylorida striata; kingdom: Animalia; phylum: Arthropoda; class: Arachnida; order: Araneae; family: Tetragnathidae; genus: Tylorida; taxonRank: species; scientificNameAuthorship: (Thorell, 1877); **Location:** continent: Asia; country: Indonesia; countryCode: IDN; stateProvince: NORTH SULAWESI; locality: (Palu) Lore Lindu Res; **Event:** eventDate: 07/23/1982; **Record Level:** type: Specimen; institutionID: Naturalis Biodiversity Center, Leiden; institutionCode: RMNH; collectionCode: Arachnida; basisOfRecord: PreservedSpecimen
- **Type status:** Other material. **Occurrence:** recordedBy: C.L and P.R. Deeleman; individualCount: 1; sex: 1 Female; **Taxon:** taxonID: urn:lsid:nmbe.ch:spidersp:014401; scientificName: Tylorida striata; kingdom: Animalia; phylum: Arthropoda; class: Arachnida; order: Araneae; family: Tetragnathidae; genus: Tylorida; taxonRank: species; scientificNameAuthorship: (Thorell, 1877); **Location:** continent: Asia; country: Indonesia; countryCode: IDN; stateProvince: SUMATRA; locality: 20 km east of Medan; **Event:** eventDate: 01/07/2001; **Record Level:** type: Specimen; institutionID: Naturalis Biodiversity Center, Leiden; institutionCode: RMNH; collectionCode: Arachnida; basisOfRecord: PreservedSpecimen
- **Type status:** Other material. **Occurrence:** recordedBy: Deeleman; individualCount: 2; sex: 1 male, 1 juvenile; **Taxon:** taxonID: urn:lsid:nmbe.ch:spidersp:014401; scientificName: Tylorida striata; kingdom: Animalia; phylum: Arthropoda; class: Arachnida; order: Araneae; family: Tetragnathidae; genus: Tylorida; taxonRank: species; scientificNameAuthorship: (Thorell, 1877); **Location:** continent: Asia; country: Indonesia; countryCode: IDN; stateProvince: SUMATRA; locality: Mount Singalang; **Event:** eventDate: 07/10/1994; **Record Level:** type: Specimen; institutionID: Naturalis Biodiversity Center, Leiden; institutionCode: RMNH; collectionCode: Arachnida; basisOfRecord: PreservedSpecimen
- **Type status:** Other material. **Occurrence:** recordedBy: leg. Preston; individualCount: 2; sex: 1 male, 1 female; **Taxon:** taxonID: urn:lsid:nmbe.ch:spidersp:014401; scientificName: Tylorida striata; kingdom: Animalia; phylum: Arthropoda; class: Arachnida; order: Araneae; family: Tetragnathidae; genus: Tylorida; taxonRank: species; scientificNameAuthorship: (Thorell, 1877); **Location:** continent: Asia; country: Indonesia; countryCode: IDN; stateProvince: SULAWESI; locality: Tangkoko; **Event:** eventDate: 02/26/1997; **Record Level:** type: Specimen; institutionID: Naturalis Biodiversity Center, Leiden; institutionCode: RMNH; collectionCode: Arachnida; basisOfRecord: PreservedSpecimen
- **Type status:** Other material. **Occurrence:** recordedBy: leg. Preston-Mafham; individualCount: 21; sex: 15 females, 6 juveniles; **Taxon:** taxonID: urn:lsid:nmbe.ch:spidersp:014401; scientificName: Tylorida striata; kingdom: Animalia; phylum: Arthropoda; class: Arachnida; order: Araneae; family: Tetragnathidae; genus: Tylorida; taxonRank: species; scientificNameAuthorship: (Thorell, 1877); **Location:** continent: Asia; country: Indonesia; countryCode: IDN; stateProvince: BALI; locality: Ambengan; **Event:** eventDate: 03/19/1990; **Record Level:** type: Specimen; institutionID: Naturalis Biodiversity Center, Leiden; institutionCode: RMNH; collectionCode: Arachnida; basisOfRecord: PreservedSpecimen
- **Type status:** Other material. **Occurrence:** recordedBy: Deeleman and leg. Suh. Djojosudharmo; individualCount: 4; sex: 3 females, 1 juvenile; **Taxon:** taxonID: urn:lsid:nmbe.ch:spidersp:014401; scientificName: Tylorida striata; kingdom: Animalia; phylum: Arthropoda; class: Arachnida; order: Araneae; family: Tetragnathidae; genus: Tylorida; taxonRank: species; scientificNameAuthorship: (Thorell, 1877); **Location:** continent: Asia; country: Indonesia; countryCode: IDN; stateProvince: SUMBAWA BESAR; locality: Samokat, 20 km west; **Event:** eventDate: 01/03/1990; **Record Level:** type: Specimen; institutionID: Naturalis Biodiversity Center, Leiden; institutionCode: RMNH; collectionCode: Arachnida; basisOfRecord: PreservedSpecimen
- **Type status:** Other material. **Occurrence:** recordedBy: Leg. M.v.D. Putten; individualCount: 3; sex: 3 juveniles; **Taxon:** taxonID: urn:lsid:nmbe.ch:spidersp:014401; scientificName: Tylorida striata; kingdom: Animalia; phylum: Arthropoda; class: Arachnida; order: Araneae; family: Tetragnathidae; genus: Tylorida; taxonRank: species; scientificNameAuthorship: (Thorell, 1877); **Location:** continent: Asia; country: Indonesia; countryCode: IDN; stateProvince: WEST IRIAN; locality: Sorong; **Event:** eventDate: 08/05/1984; **Record Level:** type: Specimen; institutionID: Naturalis Biodiversity Center, Leiden; institutionCode: RMNH; collectionCode: Arachnida; basisOfRecord: PreservedSpecimen
- **Type status:** Other material. **Occurrence:** recordedBy: Deeleman and leg. Suh. Djojosudharmo; individualCount: 3; sex: 2 females,1 juvenile; **Taxon:** taxonID: urn:lsid:nmbe.ch:spidersp:014401; scientificName: Tylorida striata; kingdom: Animalia; phylum: Arthropoda; class: Arachnida; order: Araneae; family: Tetragnathidae; genus: Tylorida; taxonRank: species; scientificNameAuthorship: (Thorell, 1877); **Location:** continent: Asia; country: Indonesia; countryCode: IDN; stateProvince: WEST SUMATRA; locality: Mount Singalang; **Event:** eventDate: 10/19-07-1994; **Record Level:** type: Specimen; institutionID: Naturalis Biodiversity Center, Leiden; institutionCode: RMNH; collectionCode: Arachnida; basisOfRecord: PreservedSpecimen
- **Type status:** Other material. **Occurrence:** recordedBy: P.R. Deeleman; individualCount: 1; sex: 1 male; **Taxon:** taxonID: urn:lsid:nmbe.ch:spidersp:014401; scientificName: Tylorida striata; kingdom: Animalia; phylum: Arthropoda; class: Arachnida; order: Araneae; family: Tetragnathidae; genus: Tylorida; taxonRank: species; scientificNameAuthorship: (Thorell, 1877); **Location:** continent: Asia; country: Indonesia; countryCode: IDN; stateProvince: EAST KALIMANTAN; locality: Sepaku; **Event:** eventDate: 07/13/1979; **Record Level:** type: Specimen; institutionID: Naturalis Biodiversity Center, Leiden; institutionCode: RMNH; collectionCode: Arachnida; basisOfRecord: PreservedSpecimen
- **Type status:** Other material. **Occurrence:** recordedBy: Deeleman; individualCount: 3; sex: 3 females; **Taxon:** taxonID: urn:lsid:nmbe.ch:spidersp:014401; scientificName: Tylorida striata; kingdom: Animalia; phylum: Arthropoda; class: Arachnida; order: Araneae; family: Tetragnathidae; genus: Tylorida; taxonRank: species; scientificNameAuthorship: (Thorell, 1877); **Location:** continent: Asia; country: Indonesia; countryCode: IDN; stateProvince: BALI; locality: Ambengan; **Event:** eventDate: 01/20/1990; **Record Level:** type: Specimen; institutionID: Naturalis Biodiversity Center, Leiden; institutionCode: RMNH; collectionCode: Arachnida; basisOfRecord: PreservedSpecimen
- **Type status:** Other material. **Occurrence:** recordedBy: Deeleman; individualCount: 1; sex: 1 female; **Taxon:** taxonID: urn:lsid:nmbe.ch:spidersp:014401; scientificName: Tylorida striata; kingdom: Animalia; phylum: Arthropoda; class: Arachnida; order: Araneae; family: Tetragnathidae; genus: Tylorida; taxonRank: species; scientificNameAuthorship: (Thorell, 1877); **Location:** continent: Asia; country: Indonesia; countryCode: IDN; stateProvince: AMBON; locality: Hila Garden; **Event:** eventDate: 01/30/1995; **Record Level:** type: Specimen; institutionID: Naturalis Biodiversity Center, Leiden; institutionCode: RMNH; collectionCode: Arachnida; basisOfRecord: PreservedSpecimen
- **Type status:** Other material. **Occurrence:** recordedBy: Deeleman and leg. Suh. Djojoosudharmo; individualCount: 3; sex: 1 male, 2 females; **Taxon:** taxonID: urn:lsid:nmbe.ch:spidersp:014401; scientificName: Tylorida striata; kingdom: Animalia; phylum: Arthropoda; class: Arachnida; order: Araneae; family: Tetragnathidae; genus: Tylorida; taxonRank: species; scientificNameAuthorship: (Thorell, 1877); **Location:** continent: Asia; country: Indonesia; countryCode: IDN; stateProvince: WEST.SUMATRA; locality: Mount Singalang; **Event:** eventDate: 07/12/1994; **Record Level:** type: Specimen; institutionID: Naturalis Biodiversity Center, Leiden; institutionCode: RMNH; collectionCode: Arachnida; basisOfRecord: PreservedSpecimen
- **Type status:** Other material. **Occurrence:** recordedBy: Deeleman and leg. Suh. Djojoosudharmo; individualCount: 12; sex: 3 males, 9 females; **Taxon:** taxonID: urn:lsid:nmbe.ch:spidersp:014401; scientificName: Tylorida striata; kingdom: Animalia; phylum: Arthropoda; class: Arachnida; order: Araneae; family: Tetragnathidae; genus: Tylorida; taxonRank: species; scientificNameAuthorship: (Thorell, 1877); **Location:** continent: Asia; country: Indonesia; countryCode: IDN; stateProvince: NORTH BALI; locality: Ambengan; **Event:** eventDate: 01/09/1990; **Record Level:** type: Specimen; institutionID: Naturalis Biodiversity Center, Leiden; institutionCode: RMNH; collectionCode: Arachnida; basisOfRecord: PreservedSpecimen

#### Description

Syntypes (Fig. 1) (examined by Siddharth Kulkarni). Two adult females, undissected epigyna, abdomen detached from cephalothorax; abdomen of one female damaged right laterally. Measurements of one syntype. Total length 5.36, carapace 2.03, 1.60 wide, abdomen 2.74 long, 3.14 high.

**Figure 2.**
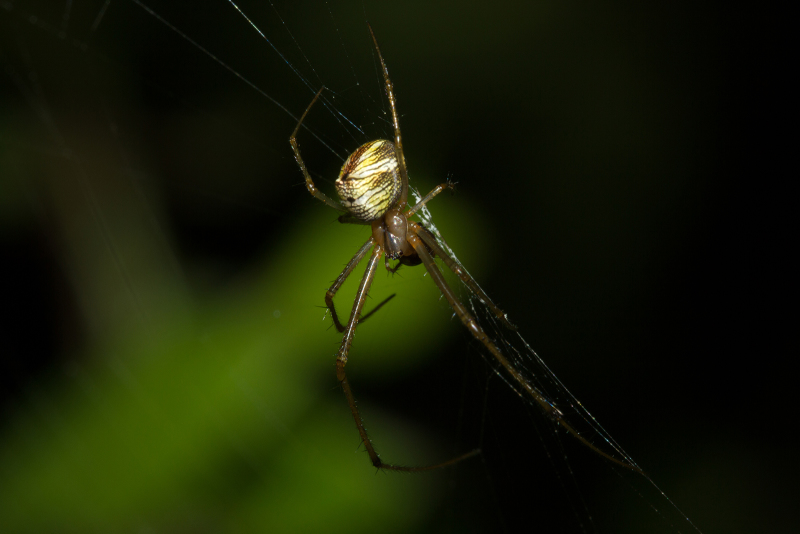
*Tylorida striata*, live female from India (BNHS Sp. 139).

Carapace brown, sternum and legs fawn; trichobothrial sockets on proximal quarter length of femora IV. Abdomen yellowish brown with shiny guanine patches lined from apex towards ventral side, as also seen in live specimen (Fig. 2). Abdomen globose, roughly triangular in lateral view with two small tubercles at apex. Epigynal plate flat, 1.5 times wider than long, coils of fertilization ducts seen translucently (Fig. 1c).

**Figure 3.**
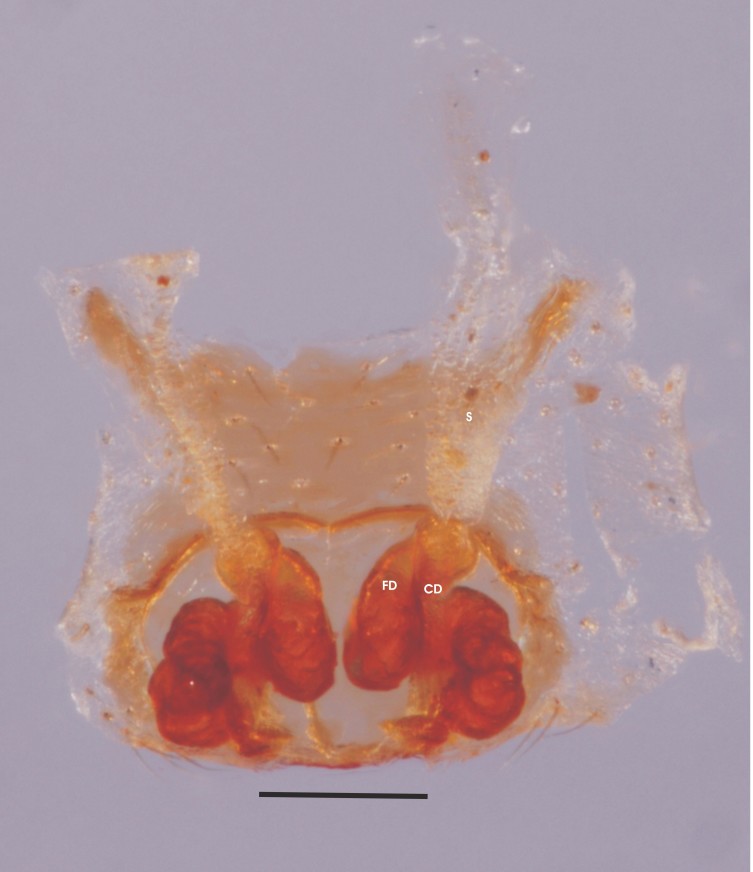
*Tylorida striata*, vulva, dorsal view, specimen from India (BNHS Sp. 139) CD- copulatory duct, FD- fertilization duct, S- spermatheca (Scale=0.2 mm).

Dissected epigynum in other material shows, coiled and sclerotized fertilization and copulatory ducts, parallel just before joining spermatheca (Fig. 3​).

##### Remarks

The female length of *T. striata* per taxonomic literature and examined specimens ranges between 3.2-5.5 mm (Suppl. material 1​). Álvarez-Padilla and Hormiga (2011) reported average size of female as 10 mm which is an error (pers. comm. Fernando Álvarez-Padilla).

##### Synonymy

Original description of *T. stellimicans* by Simon (1885)​ mentioned 'excessivement voisine' (extremely close) to *T. striata* distinguished *T. stellimicans* on the basis of arrangement and size of eyes. This description is of sub-adult *T. striata* where eye dimensions vary and ventral side of abdomen including epigynal plate surface is blackish. Also, wide distribution of *T. striata* including locality region of *T. stellimicans* and apparent absence of records elsewhere of *T. stellimicans*; we propose *T. stellimicans* as synonym of *T. striata*.

#### Taxon discussion

Dimitrov et al. (2008) stated the location of type material was uncertain and possibly in the Natural History Museum of the city of Geneva based on unpublished notes of Levi. The type(s) of *T. striata* are not there and none of Thorell’s types have been deposited in Geneva (pers. comm. Peter Schwendinger, Curator, Natural History Museum, Geneva). There is likely to be confusion between Geneva (Switzerland) and Genoa (Italy) due to similar spellings and pronunciation. Many of Thorell’s types have been deposited at Museo Civico di Storia Naturale di Genova (also known as the Natural History Museum of Giacomo Doria, Genoa). The existence of two syntypes collected by O. Beccari in 1874 from Kandari, Indonesia was confirmed (Fig. 1d) at this museum in Genoa. Thorell (1877) mentions collection of three females, but only two were found in the vial labelled as ‘Cotypi’ and one remaining specimen is missing.

## Discussion

**Figure 4.**
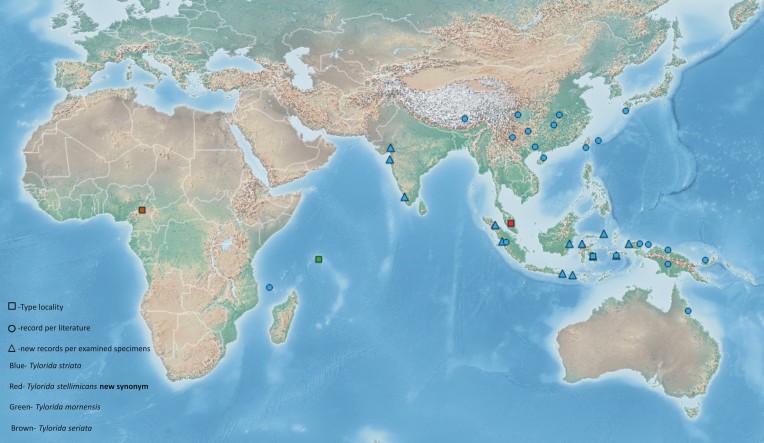
Map showing distribution of *Tylorida* species with *T. striata*-like globose abdomen.

The new record of *T. striata* from the Indian peninsula joins the disjunction of this species’ distribution between Comoros Islands and China to Australia (Fig. 4). Interestingly, the species is found on either sides of the Wallace line, thus distributed throughout the two ecozones of Asia (Indo-Malayan & Palearctic) and Australasia. The record from Comoros Islands, which belongs to the Afrotropical ecozone is noteworthy. The wide distribution of *T. striata* could be attributed to the dispersal and adaptive capacity of this species. On the other hand, *Tylorida sataraensis* Kulkarni, 2014 is restricted to a smaller part of Western Ghats in India (Kulkarni and Lewis 2015). These distributional variations of species belonging to same genus may suggest contrasting adaptive capacities, since *T. striata* undoubtedly sustains in anthropogenically exploited habitats whereas, *T. sataraensis* is a habitat specialist, currently under threat of habitat degradation (Kulkarni 2014, Kulkarni and Lewis 2015). Two other *Tylorida* species having *T. striata*-like globose abdomen, *Tylorida mornensis* (Benoit, 1978) and *Tylorida seriata* (Thorell, 1899) are only known from their respective type localities (Fig. 4). *Tylorida seriata* has not been reported since its original description from Cameroon whereas *T. mornensis* was recorded again from its type locality Seychelles and reported as endemic (Saaristo 2003, Saaristo 2010​). Whether this is a natural disjunction, or due to lack of study (Dimitrov et al. 2008​) or a case of synonymy similar to *T. stellimicans* needs to be resolved, subject to a future genus revision.

## Supplementary Material

Supplementary material 1 Female length of Tylorida striata per taxonomic reference, examined syntypes and specimens from India Data type: Morphological
File: oo_39588.xlsx Siddharth Kulkarni and Swara Yadav

XML Treatment for Tylorida striata

## Figures and Tables

**Figure 1a.**
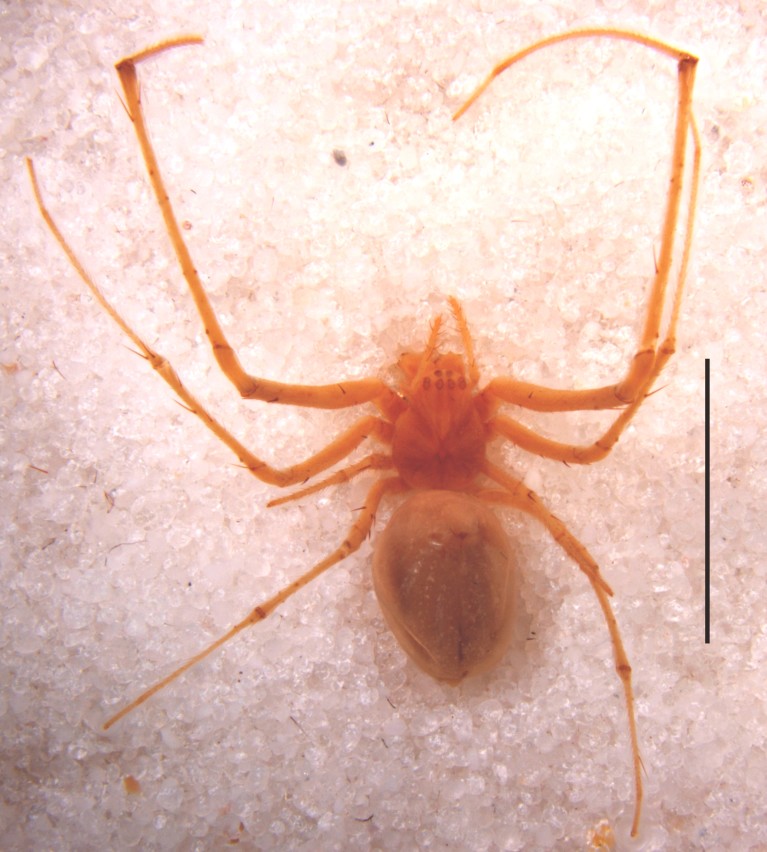
Habitus (detached cephalothorax and abdomen placed together), dorsal view (Scale=5 mm)

**Figure 1b.**
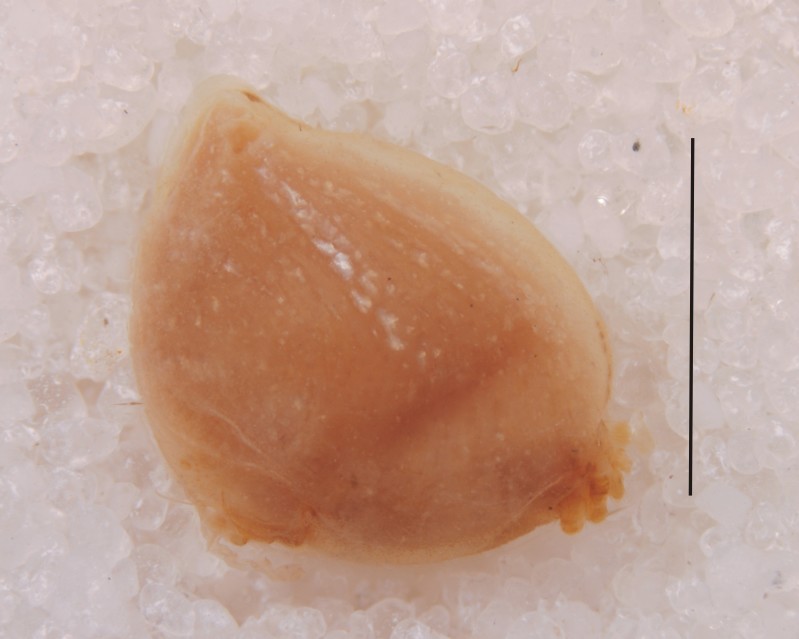
Abdomen of same syntype, lateral view (Scale=3 mm)

**Figure 1c.**
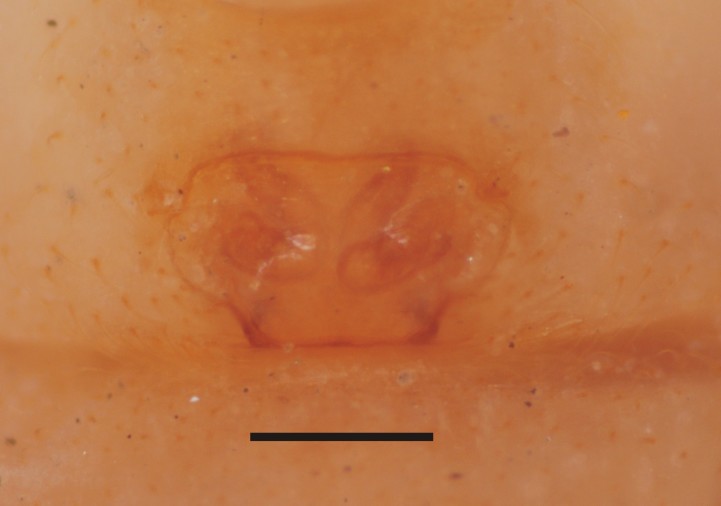
Vulva, ventral view (Scale=0.3 mm)

**Figure 1d.**
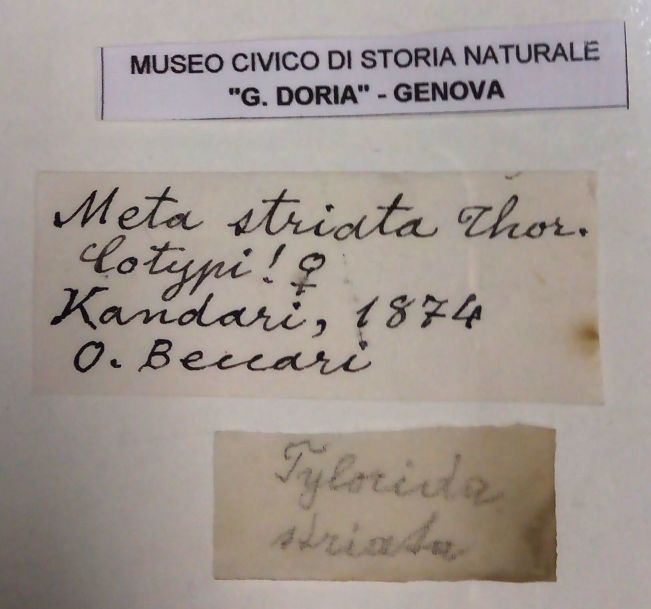
Labels in syntype vial
